# Effects of Meteorological Factors and Atmospheric Pollution on Hand, Foot, and Mouth Disease in Urumqi Region

**DOI:** 10.3389/fpubh.2022.913169

**Published:** 2022-06-22

**Authors:** Fang-rong Ren, Yakup Abodurezhake, Zhe Cui, Miao Zhang, Yu-yu Wang, Xue-rong Zhang, Yao-qin Lu

**Affiliations:** ^1^College of Economics and Management, Nanjing Forestry University, Nanjing, China; ^2^College of Public Health, Xinjiang Medical University, Urumqi, China; ^3^Economics and Management School, Nantong University, Nantong, China; ^4^Department of Infectious Disease Control, Urumqi Center for Disease Control and Prevention, Ürümqi, China

**Keywords:** meteorological factor, atmospheric pollution, multiple regression model, prevention of HFMD, communicable disease control

## Abstract

**Background:**

Hand, foot, and mouth disease (HFMD) is a febrile rash infection caused by enteroviruses, spreading mainly via the respiratory tract and close contact. In the past two decades, HFMD has been prevalent mainly in Asia, including China and South Korea, causing a huge disease burden and putting the lives and health of children at risk. Therefore, a further study of the factors influencing HFMD incidences has far-reaching implications. In existing studies, the environmental factors affecting such incidences are mainly divided into two categories: meteorological and air. Among these studies, the former are the majority of studies on HFMD. Some scholars have studied both factors at the same, but the number is not large and the findings are quite different.

**Methods:**

We collect monthly cases of HFMD in children, meteorological factors and atmospheric pollution in Urumqi from 2014 to 2020. Trend plots are used to understand the approximate trends between meteorological factors, atmospheric pollution and the number of HFMD cases. The association between meteorological factors, atmospheric pollution and the incidence of HFMD in the Urumqi region of northwest China is then investigated using multiple regression models.

**Results:**

A total of 16,168 cases in children are included in this study. According to trend plots, the incidence of HFMD shows a clear seasonal pattern, with O_3_ (ug/m^3^) and temperature (°C) showing approximately the same trend as the number of HFMD cases, while AQI, PM_2.5_ (ug/m^3^), PM_10_ (ug/m^3^) and NO_2_ (ug/m^3^) all show approximately opposite trends to the number of HFMD cases. Based on multiple regression results, O_3_ (*P* = 0.001) and average station pressure (*P* = 0.037) are significantly and negatively associated with HFMD incidences, while SO_2_ (*P* = 0.102), average dew point temperature (*P* = 0.072), hail (*P* = 0.077), and thunder (*P* = 0.14) have weak significant relationships with them.

## Introduction

Hand, foot, and mouth disease (HFMD) is a febrile rash infection caused by enteroviruses and is mainly spread through the respiratory tract and close contact. Many enteroviruses can cause HFMD, among which enterovirus 71 (ntervius71, EV71) and coxsackievirus group A type 16 (CVA16) infections are the most significant and common (1, 2). Most clinical manifestations of HFMD are mild, but sometimes EV71 infection has a chance of serious complications, including aseptic meningitis and neurological pulmonary edema, which are the main causes of death (1, 3).

In 1957 HFMD was identified in New Zealand, and since then it has caused multiple outbreaks and millions of illnesses globally (4). This indicates that HFMD has gradually become an urgent global public health problem. China is one of the countries with a higher reported morbidity and mortality from HFMD worldwide. In 1981, China first reported HFMD's appearance in Shanghai, and since then, HFMD has been reported in more than 10 provinces, such as Beijing, Hebei, Tianjin, and so on. *The Guidelines for Prevention and Control of HFMD (2008 Edition)* issued by the National Health Commission of the People's Republic of China showed that an outbreak of HFMD caused by CVA16 occurred in Tianjin in 1983, with more than 7,000 cases during May to October; in 1998, an epidemic of HFMD caused by EV71 infection occurred in Taiwan, with a total of 129,106 cases reported by sentinels surveillance.

Because HFMD is highly contagious and prone to outbreaks or epidemics, China has classified it as a Class C infectious disease for management since May 2, 2008. According to the *Pilot Operation Program for Hand, Foot and Mouth Disease Surveillance* issued by the National Health Commission of the People's Republic of China, HFMD has ranked in the top 5 in terms of the number of incidences and deaths of legally reported infectious diseases nationwide since 2009, putting many children's lives and health in danger. Although a HFMD vaccine against EV71 was marketed in China in 2016, which significantly reduced the morbidity and mortality of HFMD, the vaccine cannot completely prevent HFMD, and the incidence rate of HFMD still hit 0.5423‰ in 2020. There is still a lack of specific therapeutic drugs for HFMD. Therefore, studying the factors affecting HFMD incidences have significance in reducing its morbidity and mortality through a better understanding of them.

From the summary of the current study, it is known that the factors influencing its incidences are mainly divided into two categories: meteorological factors and air pollutants. Within the literature, meteorological factors make up the majority of studies on HFMD, but the results of meteorological factors in different studies are not identical. Most studies have demonstrated significant relationships among relative humidity, mean temperature, precipitation, and sunshine hours and HFMD incidences (5–7). However, in Lau et al. (8) and Zhao et al. (9), no statistically significant relationship appears between precipitation and such incidences, which is different from the findings of previous studies. This difference can be attributed to local weather conditions, demographic characteristics, and socioeconomic factors. In studies on wind speed, the results all showed an association with HFMD incidences, but there is a big controversy in terms of significance. Unlike scholars as Liao et al. (10) and Tian et al. (11) who considered the effect of wind speed to be significant, Xu et al. (12) and Zheng et al. (13) had the opposite conclusion that these incidences lacked significant correlation with wind speed. In addition, the number of studies involving barometric pressure, dew point temperature, and horizontal visibility is low, and so this present study investigates meteorological factors such as average station pressure, average dew point temperature, hail, and thunder.

It is worth mentioning that although there are fewer articles individually focusing on the influence of atmospheric pollution on HFMD incidences (14–17), the studies have mostly reached the same conclusion. Although different scholars have investigated how atmospheric pollution factors vary, the results obtained are that the atmospheric pollution factors do influence such incidences. Since both atmospheric pollution and meteorological factors may affect HFMD incidences and there may be potential interactions between them (17), it is important to investigate these factors' influence by simultaneously studying the effects of atmospheric pollution and meteorological conditions. Although some scholars have studied both factors at the same, the number is not large, and the findings are quite different. For example, Huang et al. (18) found that HFMD incidences are significantly associated with temperature, but not PM_10_, while Zhong et al. (19) concluded that both temperature and air quality are associated with HFMD outbreaks and that improving air quality, especially reducing PM_2.5_ and PM_10_, could reduce the risk of HFMD outbreaks. The reason for this difference can be blamed for the different geography of the study area, especially the different latitudes, making the interactions between the influencing factors inconsistent, which leads to results that differ in terms of the degree of impact on HFMD incidences.

It is not difficult to find that most existing studies have focused on tropical or coastal areas of the Asia-Pacific region, while fewer studies have been conducted on places with variable and complex environments. For example, Huang et al. (20) conducted their survey on Ningbo, one of China's landlocked regions. The northwestern part of China is located in the hinterland of the Asia-Europe continent, is deep inland, and has scarce precipitation throughout the year and a continental climate. The terrain is undulating and topographically complex. Therefore, this paper takes Urumqi in northwest China as the study area.

Based on the daily data of meteorological factors such as average station pressure, average dew point temperature, hail, and thunder, and daily data of atmospheric pollution such as O_3_ and SO_2_ in Urumqi from 2014 to 2020, this paper uses multiple regression models to investigate the factors influencing the incidences of HFMD over these 7 years. It analyzes the effects of atmospheric pollution and climatic factors on such incidences in northwestern China simultaneously for the very time, offering important implications for reducing them in western China, as well as for other regions with similar natural and social environments, such as the central region of the United States and Afghanistan. Besides meteorological factors and atmospheric pollutants, experts such as Gao et al. (21), Liu et al. (22), and Sun et al. (23) have also started to gradually focus on the influence of socioeconomic factors on HFMD in recent years, providing ideas for further development of our study.

## Data and Methods

### Study Area

**Figure 1:**
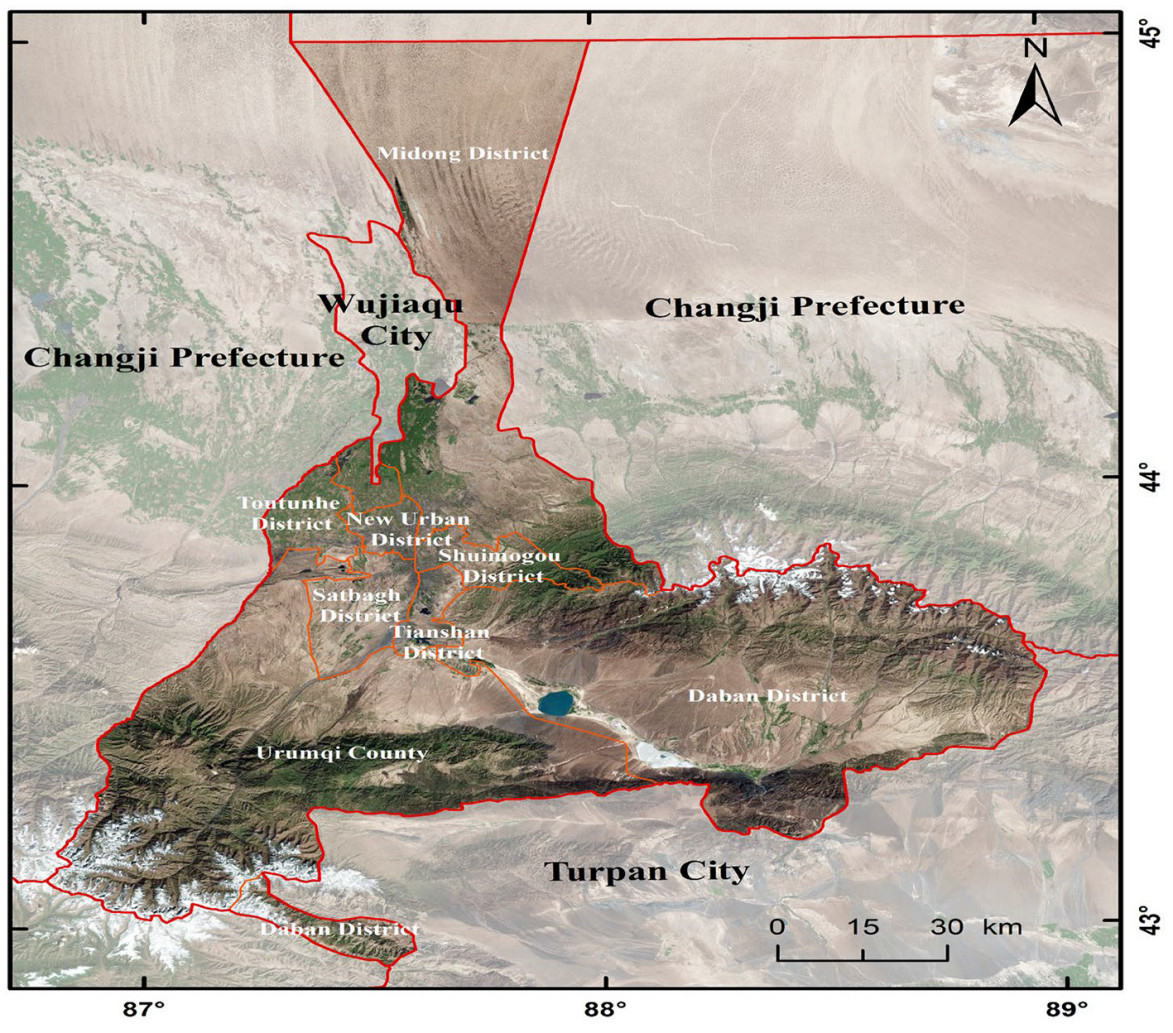
Geographical location of Urumqi City, China.

Located in northwest China, Urumqi is the farthest city in the world from any ocean and is home to a large number of ethnic minorities. Urumqi has a total area of 13,800 square kilometers and a resident population of 4.05 million (in 2020)^1^. Urumqi has a moderate temperate continental arid climate with abundant sunshine and scarce precipitation. The annual sunshine duration is 2,719.4 h, and the annual average precipitation is 194.3 mm. Urumqi has a large temperature difference between day and night. The hottest month is July and August, with an average temperature of 25.7°C; the coldest month is January, with an average temperature of −15.2°C. The northwest wind blows all year round, with an average annual temperature of 8.7°C and an average annual relative humidity of 51%. Figure 1 shows the geographical location of Urumqi.

### Data Source

Most patients with HFMD have mild symptoms, mainly fever, oral herpes or ulcers, and rashes on the palms and soles of the feet. However, a small number of patients may exhibit severe manifestations such as aseptic meningitis, brainstem encephalitis, encephalomyelitis, acute flaccid paralysis, neurogenic pulmonary hemorrhage or pulmonary edema, and cardiopulmonary failure. HFMD was included in the management of category C infectious diseases in China in 2008. All suspected or confirmed HFMD cases should be reported to the system by medical institutions, mainly hospitals, within 24 h, and all reported data are reviewed by professionals to ensure their accuracy and reliability. The data are obtained from the China Information System for Disease Control and Prevention, 2014–2020 HFMD information in the Urumqi region.

### Atmospheric Pollution and Meteorological Data

Data on meteorological factors of Urumqi from 2014 to 2020 (including temperature, barometric pressure, relative humidity, wind speed, horizontal visibility, etc.) are obtained from the UK Meteorology Office weather website (https://rp5.ru/Weather_in_the_world). The data of ambient air quality index (AQI), sulfur dioxide (SO_2_), nitrogen dioxide (NO_2_), ground-level ozone (O_3_), particulate matter (PM) with an aerodynamic diameter ≤ 10 μ (PM_10_), PM with an aerodynamic diameter ≤ 2.5 μ (PM_2.5_), and carbon monoxide (CO) from 2014 to 2020 are obtained from the General Station of China Environmental Monitoring Station, and these data are available through the national real-time air quality release platform (http://www.cnemc.cn/) and the China Air Quality Online Monitoring and Analysis Platform (https://www.aqistudy.cn/historydata/). For meteorological factors, atmospheric temperature, mean meteorological station horizontal pressure, mean sea level pressure, relative humidity, and dew point temperature are observed at a height of 2 m above ground level, and wind speed is the average wind speed at a height of 10–12 m above ground level during the 10 min before the observation. Horizontal visibility is influenced by solid and liquid particles in the air and is an important indicator of the quality of the atmospheric environment. Horizontal visibility in this study is measured by transmission and scattering visibility instruments.

## Results

In the present study, the total number of children aged 0–15 years with HFMD reached 16,168. Table 1 summarizes the basic information about the number of HFMD cases, atmospheric pollution, and meteorological factors. The monthly average values of the number of HFMD cases in Urumqi are 193 (range: 1–1,331). The monthly average values of atmospheric pollution factors such as AQI, PM_2.5_, PM_10_, NO_2_, and O_3_ are 102.1 (range: 56.0–277.2), 62.5 ug/m^3^ (range: 13.3–237.8 ug/m^3^), 117.3 ug/m^3^ (range: 30.5–318.9 ug/m^3^), 46.5 ug/m^3^ (range: 11.4–89.1ug/m^3^), and 49.0 ug/m^3^ (range: 7.6–117.0 ug/m^3^), respectively. In addition, the monthly average values of meteorological factors such as temperature and relative humidity are 8.3°C (range: −16.4 to 26.8°C) and 55.7% (range: 28.8–84.9%), respectively.

**Table 1:** Description of HFMD cases and meteorological and atmospheric pollution factors in Urumqi from 2014 to 2020.

**Variable**	**Min**	**Max**	**Mean ±SD**	**P1**	**P10**	**Median**	**P90**	**P99**
Case	1.0	1,331.0	192.5 ± 271.4	1.8	6.9	89.5	503.4	1,136.8
AQI	56.0	277.2	102.1 ± 48.3	56.1	63.1	82.3	171.2	250.5
PM_2.5_ (ug/m^3^)	13.3	237.8	62.5 ± 50.8	14.3	19.3	41.5	137.0	215.6
PM_10_ (ug/m^3^)	30.5	318.9	117.3 ± 56.8	38.6	66.2	109.5	202.3	295.3
SO_2_ (ug/m^3^)	5.7	51.9	13.8 ± 9.7	5.8	7.3	9.5	27.1	50.3
NO_2_ (ug/m^3^)	11.4	89.1	46.5 ± 16.5	18.9	30.1	43.6	69.9	87.4
O_3_ (ug/m^3^)	7.6	117.0	49.0 ± 28.9	7.8	15.7	44.5	87.2	115.4
CO (mg/m^3^)	0.5	3.5	1.3 ± 0.8	0.5	0.6	0.9	2.6	3.4
High temperature (°C)	−12.2	31.7	12.8 ± 13.6	−11.2	−6.7	15.7	28.8	31.6
Low temperature (°C)	−19.0	21.4	3.8 ± 12.3	−17.5	−13.7	5.7	18.6	21.2
Temperature (°C)	−16.4	26.8	8.3 ± 13.1	−15.6	−10.4	10.1	23.9	26.8
Relative humidity (%)	28.8	84.9	55.7 ± 16.6	30.9	36.1	49.0	78.3	83.5
Dew point temperature (°C)	−19.7	11.6	−2.0 ± 8.2	−18.5	−13.5	−1.5	8.5	10.0
Horizontal visibility (km)	1.5	30.0	11.3 ± 8.4	2.0	2.7	8.8	25.7	30.0
Average wind velocity (m/s)	1.3	3.1	2.0 ± 0.4	1.3	1.5	2.1	2.5	2.9
Mean sea level pressure (mmHg)	752.2	780.6	766.1 ± 8.4	752.6	755.3	765.1	777.1	780.4
Horizontal pressure of weather station (mmHg)	675.9	691.1	683.9 ± 4.0	676.5	678.3	684.3	688.6	690.8

*SD, standard deviation; Px, percentile of the data*.

### Trend Analysis of the Number of HFMD Cases and Meteorological Elements in Urumqi

**Figure 2:**
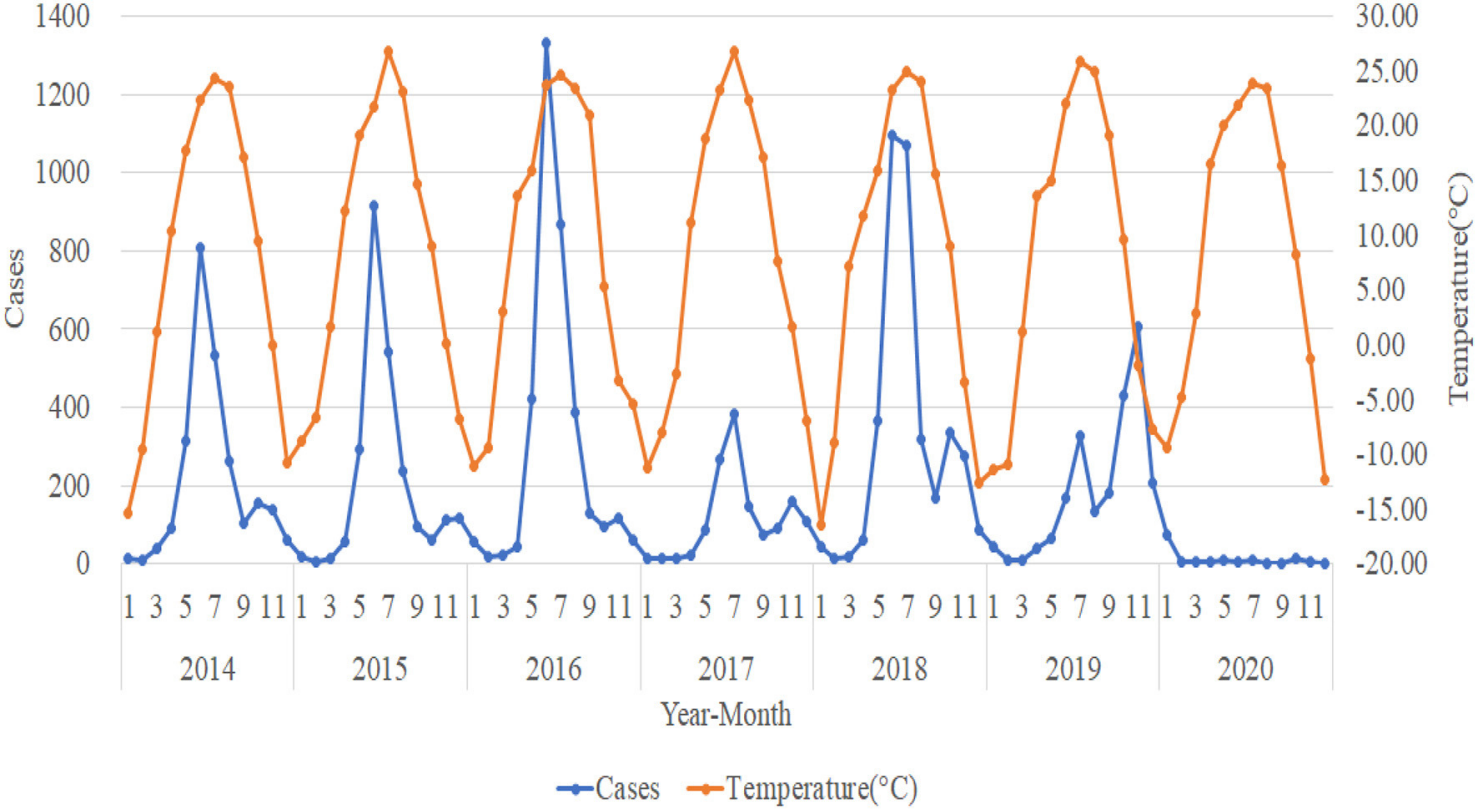
Trends in the number of HFMD cases and temperature (°C).

**Figure 3:**
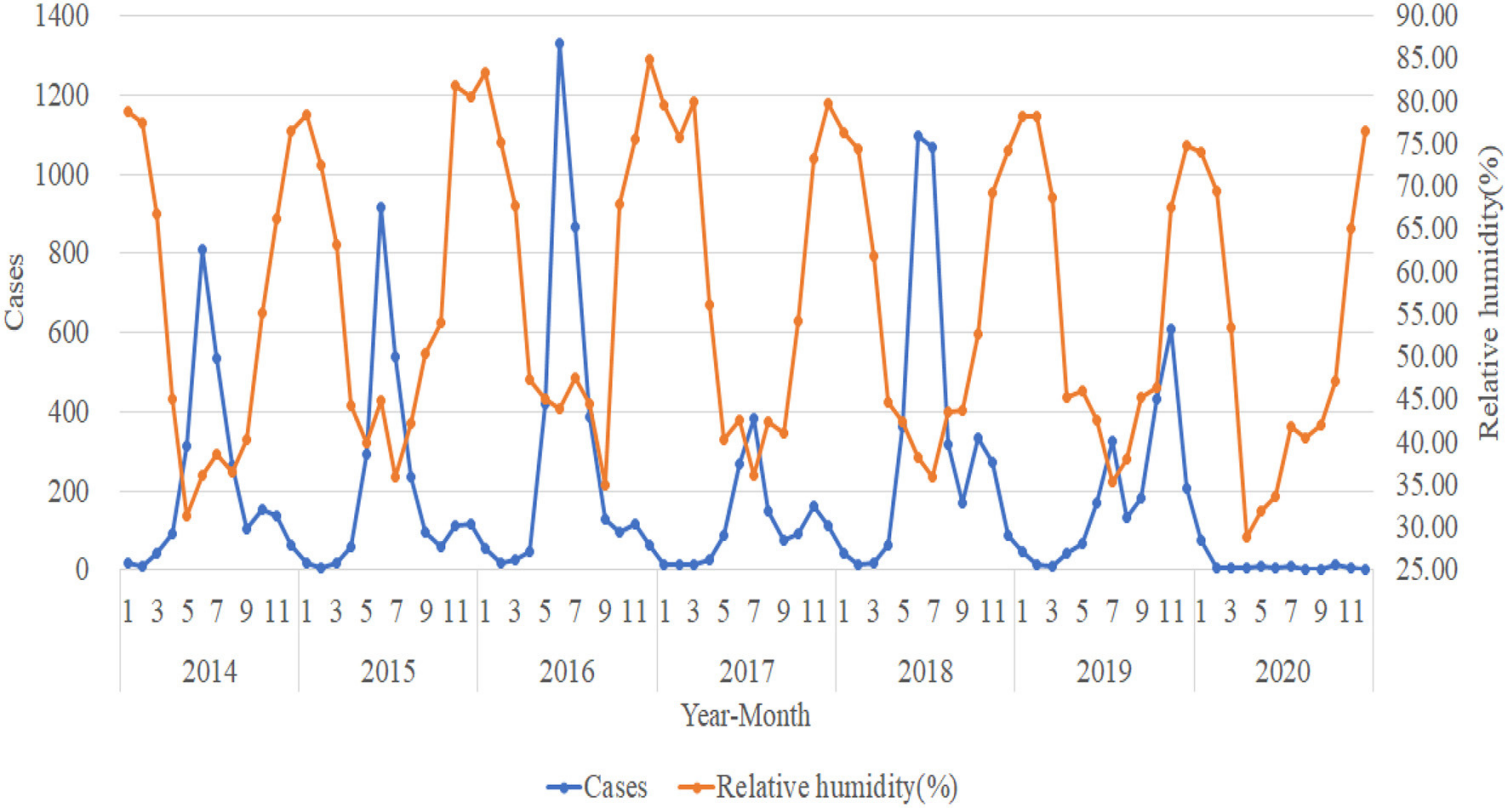
Trends in the number of HFMD cases and relative humidity (%).

This study compares the monthly HFMD cases from 2014 to 2020 with high temperature (°C), low temperature (°C), temperature (°C), relative humidity (%), dew point temperature (°C), horizontal visibility (km), average wind velocity (m/s), mean sea level pressure (mmHg), and horizontal pressure of weather station (mmHg) for plotting and trend analysis. The analysis shows that the trend in the number of HFMD cases is not significantly associated with horizontal visibility (km), average wind velocity (m/s), mean sea level pressure (mmHg), and horizontal pressure of weather station (mmHg) (not shown in this paper due to space limitation). The trends of high temperature (°C), low temperature (°C), dew point temperature (°C), and temperature (°C) are approximately the same. Thus, we select the representative trend of temperature (°C) for specific elaboration. Figures 2, 3 shows the trends in the number of HFMD cases with temperature (°C) and relative humidity (%) respectively.

The incidences of HFMD from 2014 to 2018 show a clear seasonal pattern, generally with important peaks in June and July and another small (albeit less pronounced) seasonal peak in November and December each year. July 2019 was a small seasonal peak in the incidence situation, while November saw another important peak, but not quite in line with the previous 5 years. The peak number of HFMD cases from 2014 to 2016 kept rising, and the number of HFMD cases in 2017 was remarkably lower than in other years. Additionally, the number of HFMD cases plummeted after January 2020, staying below 15 cases per month. The trend of temperature (°C) is roughly the same as the trend in the number of HFMD cases. The highest temperature month of the year correlates to the high incidences of HFMD, and when the temperature gradually increases, the number of HFMD cases also gradually increases. Accordingly, the number of HFMD cases shows the tendency of decreases as the temperature grows. The trend of relative humidity (%) is the opposite to the trend of the number of HFMD cases. When relative humidity (%) is low, the number of HFMD cases is higher, and accordingly, when relative humidity (%) is high, the number of HFMD cases is lower.

### Trend Analysis in the Number of HFMD Cases and Atmospheric Pollution Elements in Urumqi

**Figure 4:**
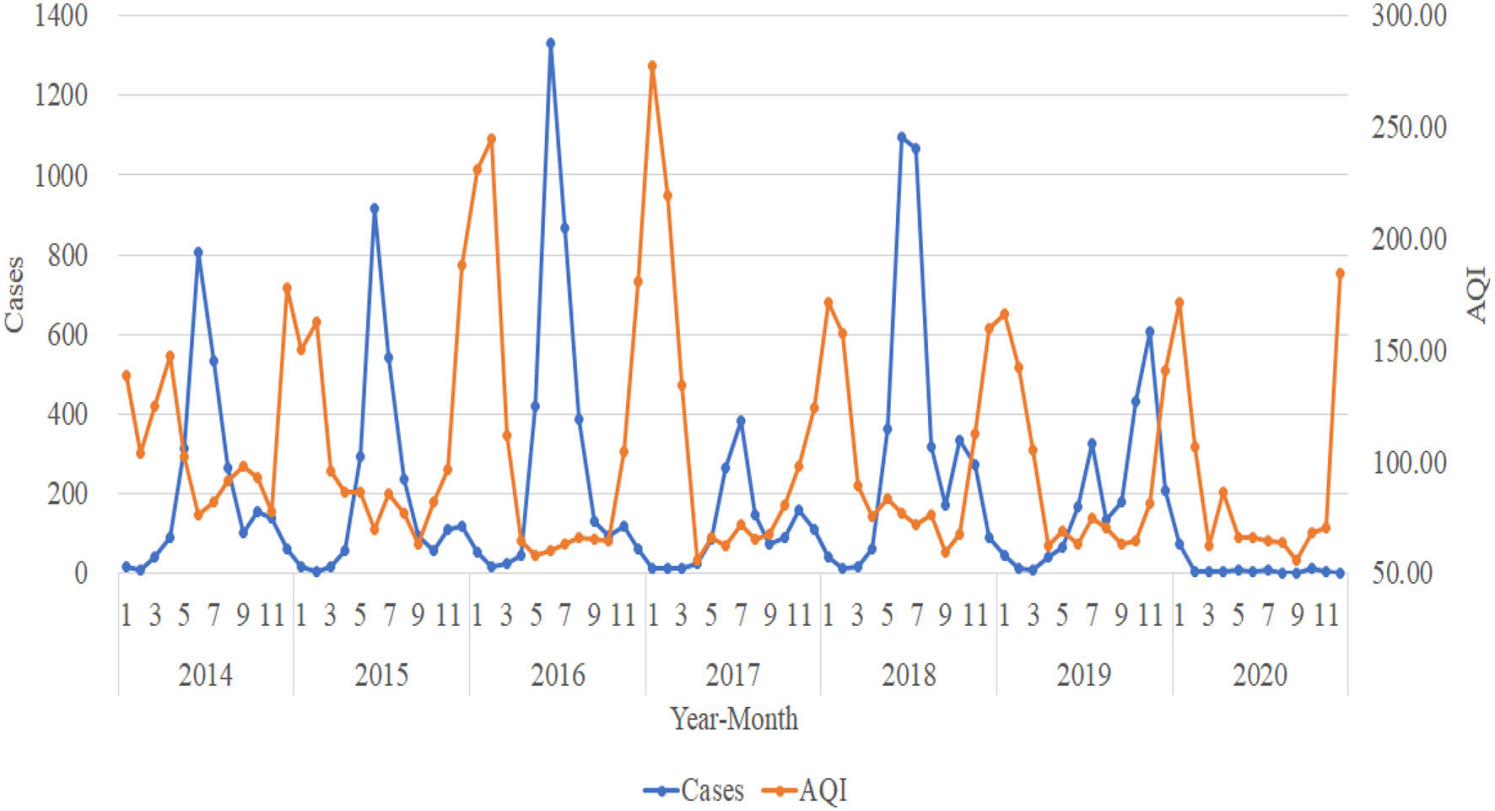
Trends in the number of HFMD cases and AQI.

**Figure 5:**
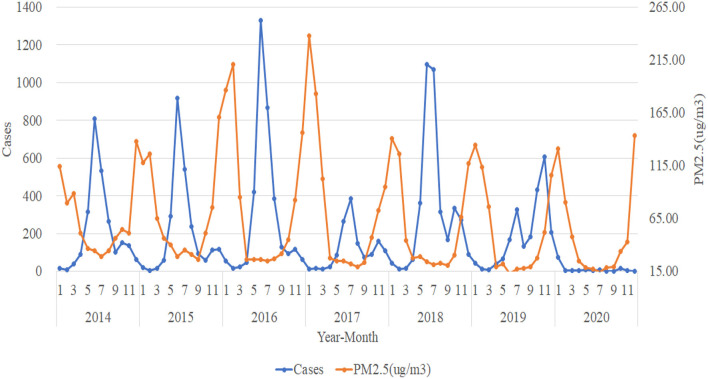
Trends in the number of HFMD cases and PM_2.5_ (ug/m^3^).

**Figure 6:**
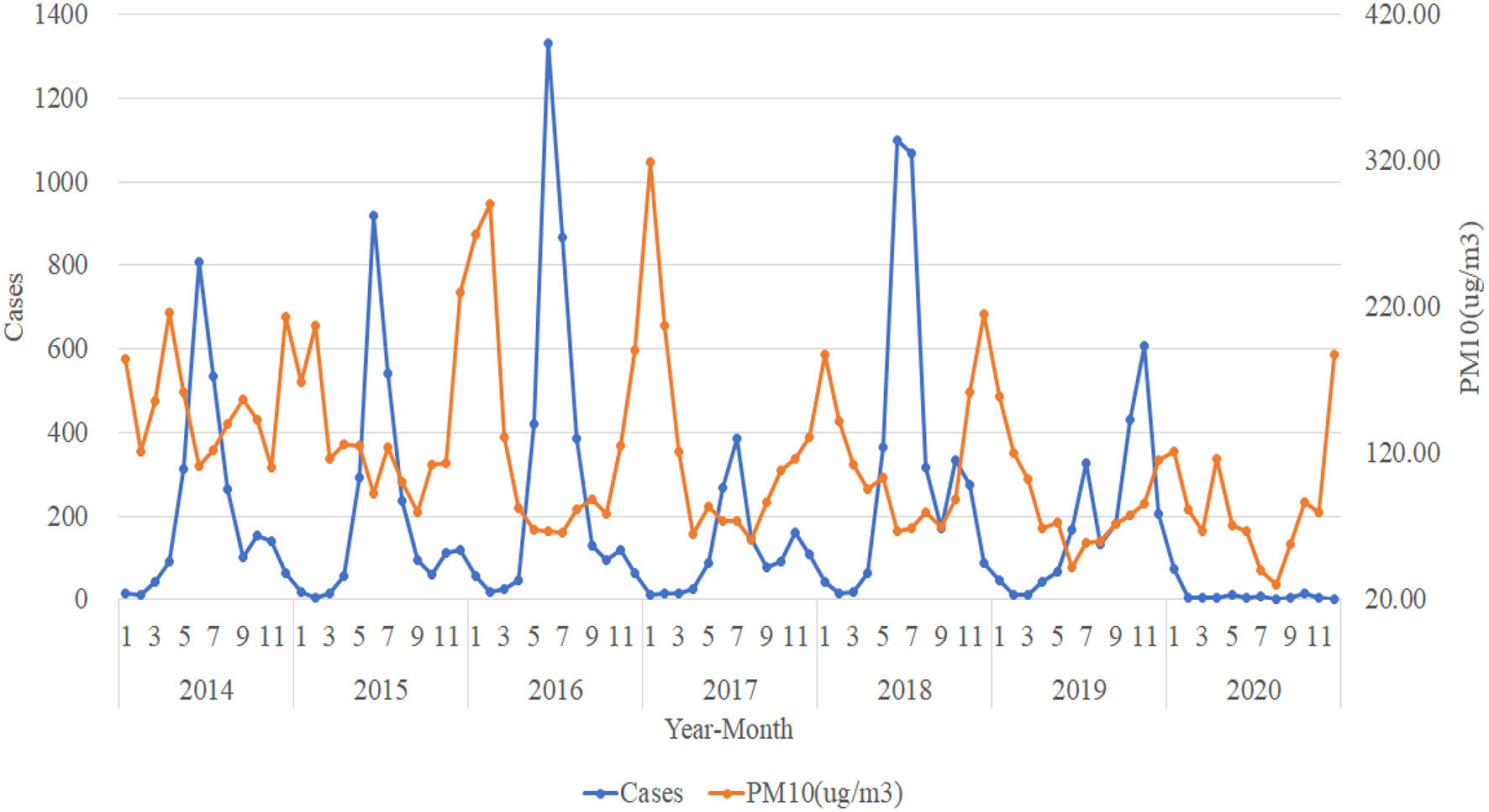
Trends in the number of HFMD cases and PM_10_ (ug/m^3^).

For the trend analysis of HFMD and atmospheric pollution, we use the same method as meteorological elements to make a plot of the number of HFMD cases per month from 2014 to 2020 with the trend of AQI, PM_2.5_ (ug/m^3^), PM_10_ (ug/m^3^), SO_2_ (ug/m^3^), NO_2_ (ug/m^3^), O_3_ (ug/m^3^), and CO (mg/m^3^). The trends of AQI, PM_2.5_ (ug/m^3^), and PM_10__(_ug/m^3^) tend to be consistent and are all approximately opposite to the trend of the number of HFMD cases. When the concentrations of AQI, PM_2.5_ (ug/m^3^), and PM_10_ (ug/m^3^) are in a relatively stable stage, the number of HFMD cases is in a rapid increase and rapid decrease stage; while when the atmospheric pollution in Urumqi is more severe and the suspended particulate matter in the air reaches higher concentrations, the number of HFMD cases gradually decreases (Figures 4–6).

**Figure 7:**
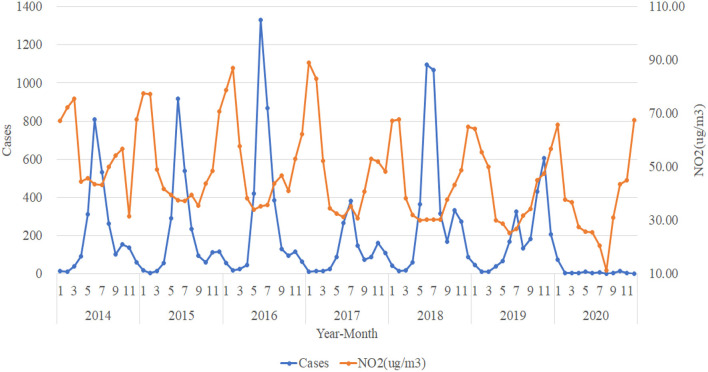
Trends in the number of HFMD cases and NO_2_ (ug/m^3^).

**Figure 8:**
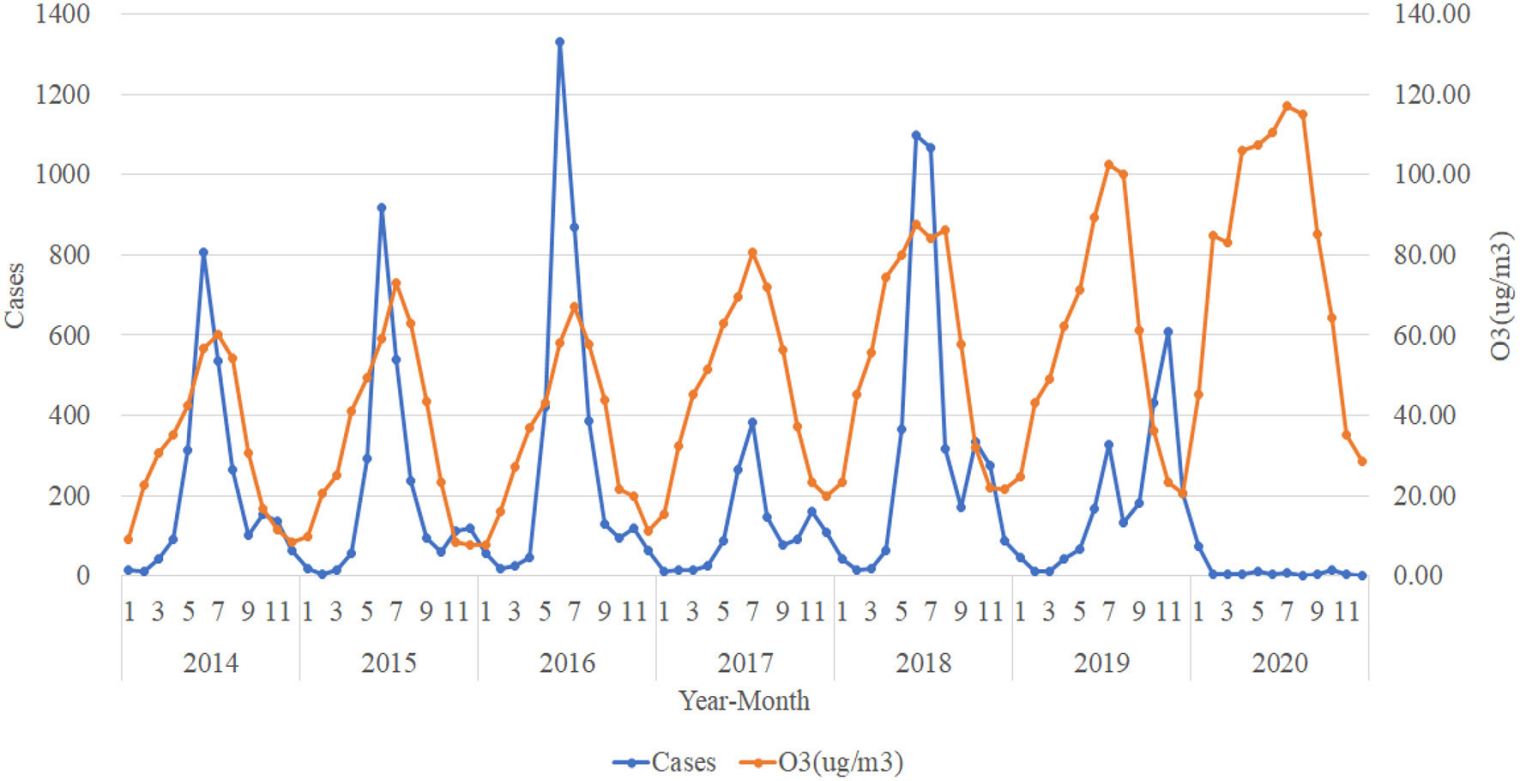
Trends in the number of HFMD cases and O_3_ (ug/m^3^).

Figure 7 shows that the trend of NO_2_ (ug/m^3^) concentration from 2014 to 2020 is roughly opposite to the trend of the number of HFMD cases, with the number of HFMD cases experiencing a peak when the concentration of NO_2_ (ug/m^3^) is relatively low and correspondingly fewer HFMD cases when the concentration of NO_2_ (ug/m^3^) is high. In contrast, the trend in the concentration of O_3_ (ug/m^3^) tends to coincide with the trend in the number of HFMD cases (Figure 8). As we can see, the trends of CO (mg/m^3^) concentration and SO_2_ (ug/m^3^) concentration do not correlate significantly with the trends of the number of HFMD cases, and so the trend change graphs of CO (mg/m^3^) and SO_2_(ug/m^3^) are not shown in this paper.

### Statistical Analysis (Regression Model for Effects of Factors on HFMD Incidences)

This study fits multiple regression models for continuous variables such as AQI, PM_2.5_, PM_10_, atmospheric temperature, and barometric pressure, in order to verify whether they work or not.

In this paper, we use the number of HFMD cases as the dependent variable and meteorological elements and atmospheric pollution elements as independent variables to build a multiple regression model with parameter estimation. However, the parameters of some of the factors were not significant, so we further conducted a multicollinearity test, we identify collinearity among factors by using variance inflation factors (VIF) and then remove individual factors that have VIF values higher than the threshold value of 10.

Finally, we optimize the model using stepwise regression, which shows that O_3_ and average station pressure are associated with the number of HFMD cases (*p* < 0.05). The regression equation is Y = 3,8025.152–4.81X_1_-39.429X_2_ (Y: Cases, X_1_: O_3_, X_2_: Average station pressure) (Table 2).

**Table 2:** Multivariate meta-regression analysis.

**Variable**	**β**	**Standard β**	**S.E**.	***t***	***P*-value**	**VIF**
(Intercept)	3,8025.152	NA	17,861.95	2.129	0.036	NA
SO_2_	−5.359	−0.192	3.241	−1.653	0.102	1.693
O_3_	−4.81	−0.512	1.38	−3.485	0.001***	2.706
Average dew point temperature	5.724	0.322	3.137	1.825	0.072*	3.896
Average station pressure	−39.429	−0.406	18.571	−2.123	0.037**	4.593
Hail	−1,7640.77	−0.163	9,852.882	−1.79	0.077*	1.034
Thunder	2,574.354	0.179	1,727.406	1.49	0.14	1.82

^*^, ^**^, and ^***^ represent significance at 0.1, 0.05, and 0.01 levels, respectively. N/A, Not applicable.

Table 2 shows the results of multivariate meta-regression analysis after removing individual factors that have VIF values higher than the threshold value of 10. The results present that O_3_ and average station pressure are remarkably and negatively associated with HFMD incidences. However, SO_2_, average dew point temperature, hail, and thunder do not significantly correlate with such incidences.

## Discussion

The effects of atmospheric pollutants and meteorological factors on human health are of great concern. Extreme weather and various atmospheric pollutants may contribute to the onset of measles (24), influenza (25–27), tuberculosis (28), COVID-19 (29, 30), and HFMD (8, 31) among other infectious diseases. HFMD has caused multiple outbreaks and millions of illnesses globally and is emerging as a pressing global public health issue (4), resulting in a significant disease burden. China is one of the countries with higher reported morbidity and mortality of HFMD worldwide. Therefore, this study uses a fitted multiple regression model and also discusses the relationship between HFMD incidences, meteorological factors, and atmospheric pollution. Understanding whether atmospheric pollution and climatic conditions affect HFMD incidences can provide a basis for the Xinjiang government to develop relevant preventive measures.

Figure 2 illustrates a rising peak in the number of HFMD cases from 2014 to 2016, which drew the attention of the relevant authorities. Thus, in February 2017 the Health Care Institute for Primary and Secondary Schools in Urumqi issued “Preventive Measures for Hand, Foot, and Mouth Disease,” which put forward seven specific requirements for how to prevent HFMD among school students in Xinjiang. In that same February, Xinjiang launched a second round of free health checkups for the whole population with increased efforts. The above two factors may have contributed to the significantly lower number of HFMD cases in 2017 than in other years. The outbreak of the COVID-19 epidemic in China in early 2020, Xinjiang's rapid activation of the Level 1 response to major public health emergencies, universal home isolation, reduced exposure, and good hygiene habits, which to some extent reduced the spread of HFMD, may have contributed to a sudden drop in the number of HFMD cases in post-January 2020.

In our study Table 2 shows that average station pressure is significantly and negatively associated with HFM incidences. HFMD is an envelope-free virus that tends to be more sensitive to changes in the environment when it survives in aerosols. When the air pressure is lower, water get diffused faster, and then the aerosol evaporates off much easier. The principle is shown below, whereby lower air pressure ensures less damage to viruses dispersed on aerosol surfaces. Surface tension shear stress and hydrophobicity can cause conformational rearrangement, which provide reasons to relatively high concentration of viruses in aerosols and increased risk of infection among people (32).

Our findings run contrary to Li et al. (33) in Guangzhou, probably due to the difference in altitude between Urumqi and Guangzhou. High-pressure provinces are positively associated with HFMD, and low-pressure provinces are negatively associated with HFMD (23). The city of Urumqi has an undulating topography with an average elevation of 800 m above sea level, high overall topography, and low average station pressure, while Guangzhou is located on the southeast coast of China with an average elevation of 11 m above sea level, low elevation, and high average station pressure. Zhao et al. (9) negated the association between barometric pressure and HFMD in a study of Huainan, Anhui, China, using a distributed lag non-linear model (DLMN), possibly due to severe covariance between weather variables and lagged effects.

Our study finds a significant negative correlation between O_3_ concentration and the incidences of HFMD, which may be due to the inhibitory effect of O_3_ on HFMD viruses. Indeed, O_3_ is a powerful airway irritant that inhibits viruses and bacteria *via* three mechanisms. First, ozone oxidizes the enzymes needed for glucose degradation in bacteria, which prevents the TCA cycle from taking place and thus prevents the supply of ATP needed for cellular life, causing inactivation and death of bacteria. Second, ozone acts directly with bacteria and viruses, destroying their organelles and DNA and RNA, so that the metabolism of bacteria is damaged, leading to the death of bacteria. Third, ozone penetrates through the cell membrane tissue and invades into the cell, acting on the lipoproteins of the outer membrane and the internal lipopolysaccharide, causing the bacteria to undergo permeability distortion and lysis death. Our results are consistent with the findings of Yan et al. (34), but they further stated that the limited protective effect of ozone on infants may be due to the limited daily activities of infants resulting in their less protection by ozone. In a study of Ningbo, China, Gu et al. (14) found that ozone concentrations are highest in summer and lower in winter, and that ozone concentrations are associated with an increased risk of HFMD, contrary to our findings, with possible explanations being that O_3_ concentrations in Ningbo are different from those in Urumqi or that other atmospheric pollutants may interact with O_3_.

In our study the relationship between SO_2_ and the incidences of HFMD is not significant, which is different from the studies of Wei et al. (15), Gu et al. (14), and Yang et al. (16). The reason for the inconsistency of our results with theirs may be the differences in regions, socioeconomic conditions, and so on.

## Advantages and Limitations

There are three major advantages of our study as follows. First, our study collected and analyzed data for a total of 7 years from 2014 to 2020, which improves the stability of the model. Second, as far as we know, this study is the first to jointly analyze the impact of atmospheric pollution and climatic factors on HFMD incidences in northwest China and provides lessons for countries and regions in the world with the same natural conditions and the same socioeconomic conditions. Finally, this paper presents significant evidence of long-term exposure to O_3_ and air pressure, which will play a very important role in follow-up studies in countries with less current research.

We have to admit that our study has a few limitations. First, not all HFMD cases are included. For example, some patients chose not to seek medical attention, because their symptoms were not obvious, and thus these cases were overlooked, leading to an underestimation in the number of HFMD cases. Second, we only considered the association between a single pollutant or a single meteorological factor and the incidences of HFMD and did not consider the mixed association of multiple pollutants and multiple meteorological factors on such incidence. Third, our study did not consider other variables such as season, socio-economic status, age, gender, etc., but only meteorological factors and atmospheric pollution, which may lead to some bias in the results. Fourth, our study is essentially ecological and can only assess the association between atmospheric pollution and meteorological factors and the incidences of HFMD, but not the specific pathways and mechanisms of HFMD from a pathological perspective. Further research is therefore needed to investigate the specific pathogenesis and mechanisms.

## Conclusion

In the last 20 years, HFMD has been prevalent mainly in the Asian region including China and South Korea and still poses a huge disease burden, so we investigate the association between meteorological factors and atmospheric pollution and HFMD incidences.

In this paper, we include a total of 16,168 cases in children in Urumqi from 2014 to 2020. The trend plots shows a clear seasonal pattern of HFMD cases, with O_3_ (ug/m^3^) and temperature (°C) showing approximately the same trend as the number of HFMD cases, while AQI, PM_2.5_ (ug/m^3^), PM_10_ (ug/m^3^) and NO_2_ (ug/m^3^) all show approximately opposite trends to the number of HFMD cases. Based on multiple regression results, O_3_ (*P* = 0.001) and average station pressure (*P* = 0.037) are significantly and negatively associated with HFMD incidences, while SO2 (*P* = 0.102), average dew point temperature (*P* = 0.072), hail (*P* = 0.077), and thunder (*P* = 0.14) have weak significant relationships with them. It demonstrates that meteorological factors and atmospheric pollution have an influential role in HFMD incidence, although the degrees of influence of different meteorological and environmental factors are not the same.

To this end, we make the following recommendations: the government and relevant authorities should pay attention to the impact of O3 and average station pressure on HFMD and remind the public to prepare in advance during the high HFMD season. In addition, the obvious seasonal high incidence difference of HFMD in Urumqi suggests that in the future HFMD prevention and control work in China, the epidemic warning of HFMD can be made in advance according to the meteorological data provided by meteorological departments in different regions, and the disease prevention work can be done well before the peak of HFMD epidemic. Timely inform nurseries, schools, medical institutions and other places where personnel are relatively concentrated to do a good job of elimination of the corresponding place, the implementation of infectious disease prevention and control measures. At the same time, due to the complex pathogenesis of HFMD, the prevention and control of HFMD should also take into account the characteristics of population flow, economic development, health education popularization and incidence treatment and other related factors, and finally get a comprehensive, accurate and timely risk assessment of HFMD. This study has important implications for reducing the incidences of HFMD in western China, as well as for the government's epidemic prediction and establishment of HFMD prevention systems to bring greater social benefits into play.

## Data Availability Statement

The original contributions presented in the study are included in the article/supplementary material, further inquiries can be directed to the corresponding author/s.

## Author Contributions

ZC: visualization, and supervision. Y-qL: methodology and data curation. F-rR: software, resources, project administration, and funding acquisition. YA: conceptualization, investigation and editing. X-rZ: validation and writing—review. MZ and Y-yW: formal analysis. MZ: writing—original draft preparation. All authors read and contributed to the manuscript.

## Conflict of Interest

The authors declare that the research was conducted in the absence of any commercial or financial relationships that could be construed as a potential conflict of interest.

## Publisher's Note

All claims expressed in this article are solely those of the authors and do not necessarily represent those of their affiliated organizations, or those of the publisher, the editors and the reviewers. Any product that may be evaluated in this article, or claim that may be made by its manufacturer, is not guaranteed or endorsed by the publisher.

## Footnotes

^1^The People's Government of Urumqi Municipality, http://www.wlmq.gov.cn.

## References

[1] Wang Y Feng Z Yang Y Self S Gao Y Longini IM. Hand, foot, and mouth disease in China: patterns of spread and transmissibility. Epidemiology. (2011) 22:781–92. 10.1097/EDE.0b013e318231d67a 21968769 PMC3246273

[2] Yan XF Gao S Xia JF Ye R Yu H Long JE. Epidemic characteristics of hand, foot, and mouth disease in Shanghai from 2009 to 2010: enterovirus 71 subgenotype C4 as the primary causative agent and a high incidence of mixed infections with coxsackievirus A16. Scand J Infect Dis. (2012) 44:297–305. 10.3109/00365548.2011.634433 22176514

[3] Singh S Chow VT Chan KP Ling AE Poh CL. RT-PCR, nucleotide, amino acid and phylogenetic analyses of enterovirus type 71 strains from Asia. J Virol Methods. (2000) 88:193–204. 10.1016/S0166-0934(00)00185-3 10960707

[4] Zhuang ZC Kou ZQ Bai YJ Cong X Wang LH Li C. Epidemiological research on hand, foot, and mouth disease in mainland China. Viruses. (2015) 7:6400–11. 10.3390/v7122947 26690202 PMC4690870

[5] Sumi A Toyoda S Kanou K Fujimoto T Mise K Kohei Y. Association between meteorological factors and reported cases of hand, foot, and mouth disease from 2000 to 2015 in Japan. Epidemiol Infect. (2017) 145:2896–911. 10.1017/S0950268817001820 28826420 PMC9152763

[6] Wu H Wang H Wang Q Xin Q Lin H. The effect of meteorological factors on adolescent hand, foot, and mouth disease and associated effect modifiers. Glob Health Action. (2014) 7:24664. 10.3402/gha.v7.24664 25098727 PMC4124175

[7] Zhang W Du Z Zhang D Yu S Hao Y. Boosted regression tree model-based assessment of the impacts of meteorological drivers of hand, foot and mouth disease in Guangdong, China. Sci Total Environ. (2016) 553:366–71. 10.1016/j.scitotenv.2016.02.023 26930310

[8] Lau SY-F Chen E Mohammad KN Cai J Wang MH Zee BC-Y. Ambient temperature and relative humidity as possible drivers of the hand, foot, and mouth disease epidemics in Zhejiang Province, China. Atmos Environ. (2021) 244:117984. 10.1016/j.atmosenv.2020.117984

[9] Zhao D Wang L Cheng J Xu J Xu Z Xie M. Impact of weather factors on hand, foot and mouth disease, and its role in short-term incidence trend forecast in Huainan City, Anhui Province. Int J Biometeorol. (2017) 61:453–61. 10.1007/s00484-016-1225-9 27557791

[10] Liao Y Ouyang R Wang J Xu B. A study of spatiotemporal delay in hand, foot and mouth disease in response to weather variations based on SVD: a case study in Shandong Province, China. BMC Public Health. (2015) 15:71. 10.1186/s12889-015-1446-6 25636654 PMC4324801

[11] Tian L Liang F Xu M Jia L Pan X Clements ACA. Spatio-temporal analysis of the relationship between meteorological factors and hand-foot-mouth disease in Beijing, China. BMC Infect Dis. (2018) 18:158. 10.1186/s12879-018-3071-3 29614964 PMC5883540

[12] Xu M Yu W Tong S Jia L Liang F Pan X. Non-linear association between exposure to ambient temperature and children's hand-foot-and-mouth disease in Beijing, China. PLoS ONE. (2015) 10:e0126171. 10.1371/journal.pone.0126171 26010147 PMC4444089

[13] Zheng S Cao CX Cheng JQ Wu YS Xie X Xu M. Epidemiological features of hand-foot-and-mouth disease in Shenzhen, China from 2008 to 2010. Epidemiol Infect. (2014) 142:1751–62. 10.1017/S0950268813002586 24139426 PMC9151244

[14] Gu S Li D Lu B Huang R Xu G. Associations between ambient air pollution and daily incidence of pediatric hand, foot and mouth disease in Ningbo, 2014-2016: a distributed lag nonlinear model. Epidemiol Infect. (2020) 148:e46. 10.1017/S0950268820000321 32127063 PMC7058833

[15] Wei Q Wu J Zhang Y Cheng Q Bai L Duan J. Short-term exposure to sulfur dioxide and the risk of childhood hand, foot, and mouth disease during different seasons in Hefei, China. Sci Total Environ. (2019) 658:116–21. 10.1016/j.scitotenv.2018.11.481 30577010

[16] Yang Z Hao J Huang S Yang W Zhu Z Tian L. Acute effects of air pollution on the incidence of hand, foot, and mouth disease in Wuhan, China. Atmos Environ. (2020) 225:117358. 10.1016/j.atmosenv.2020.117358 PMC7013846 31936369

[17] Yin F Ma Y Zhao X Lv Q Liu Y Li X. Analysis of the effect of PM10 on hand, foot and mouth disease in a basin terrain city. Sci Rep. (2019) 9:3233. 10.1038/s41598-018-35814-5 30824722 PMC6397224

[18] Huang R Bian G He T Chen L Xu G. Effects of meteorological parameters and PM10 on the incidence of hand, foot, and mouth disease in children in China. Int J Environ Res Public Health. (2016) 13:481. 10.3390/ijerph13050481 27171104 PMC4881106

[19] Zhong R Wu Y Cai Y Wang R Zheng J Lin D. Forecasting hand, foot, and mouth disease in Shenzhen based on daily level clinical data and multiple environmental factors. Biosci Trends. (2018) 12:450–5. 10.5582/bst.2018.01126 30473551

[20] Huang R Wei J Li Z Gao Z Mahe M Cao W. Spatial-temporal mapping and risk factors for hand foot and mouth disease in northwestern inland China. PLoS Negl Trop Dis. (2021) 15:e0009210. 10.1371/journal.pntd.0009210 33760827 PMC8021183

[21] Gao Y Wang H Yi S Wang D Ma C Tan B. Spatial and temporal characteristics of hand-foot-and-mouth disease and their influencing factors in Urumqi, China. Int J Environ Res Public Health. (2021) 18:4919. 10.3390/ijerph18094919 34063073 PMC8124546

[22] Liu H Song G He N Zhai S Song H Kong Y. Spatial-temporal variation and risk factor analysis of hand, foot, and mouth disease in children under 5 years old in Guangxi, China. BMC Public Health. (2019) 19:1491. 10.1186/s12889-019-7619-y 31703735 PMC6842152

[23] Sun J Wu S Yan Z Li Y Yan C Zhang F. Using geographically weighted regression to study the seasonal influence of potential risk factors on the incidence of HFMD on the Chinese mainland. ISPRS Int J Geoinform. (2021) 10:448. 10.3390/ijgi10070448

[24] Peng L Zhao X Tao Y Mi S Huang J Zhang Q. The effects of air pollution and meteorological factors on measles cases in Lanzhou, China. Environ Sci Pollut Res Int. (2020) 27:13524–33. 10.1007/s11356-020-07903-4 32030582

[25] Meng Y Lu Y Xiang H Liu S. Short-term effects of ambient air pollution on the incidence of influenza in Wuhan, China: a time-series analysis. Environ Res. (2021) 192:110327. 10.1016/j.envres.2020.110327 33075359

[26] Toczylowski K Wietlicka-Piszcz M Grabowska M Sulik A. Cumulative effects of particulate matter pollution and meteorological variables on the risk of influenza-like illness. Viruses. (2021) 13:556. 10.3390/v13040556 33810283 PMC8065612

[27] Zhang R Meng Y Song H Niu R Wang Y Li Y. The modification effect of temperature on the relationship between air pollutants and daily incidence of influenza in Ningbo, China. Respir Res. (2021) 22:153. 10.1186/s12931-021-01744-6 34016093 PMC8138986

[28] Wang W Guo W Cai J Guo W Liu R Liu X. Epidemiological characteristics of tuberculosis and effects of meteorological factors and air pollutants on tuberculosis in Shijiazhuang, China: a distribution lag non-linear analysis. Environ Res. (2021) 195:110310. 10.1016/j.envres.2020.110310 33098820

[29] Cole MA Ozgen C Strobl E. Air pollution exposure and Covid-19 in Dutch municipalities. Environ Resour Econ. (2020) 76:581–610. 10.1007/s10640-020-00491-4 32836849 PMC7399597

[30] Ma Y Zhao Y Liu J He X Wang B Fu S. Effects of temperature variation and humidity on the death of COVID-19 in Wuhan, China. Sci Total Environ. (2020) 724:138226. 10.1016/j.scitotenv.2020.138226 32408453 PMC7142681

[31] Yu G Li Y Cai J Yu D Tang J Zhai W. Short-term effects of meteorological factors and air pollution on childhood hand-foot-mouth disease in Guilin, China. Sci Total Environ. (2019) 646:460–70. 10.1016/j.scitotenv.2018.07.329 30056233

[32] Jiang Y Xu J Lai H Lin H. Association between meteorological parameters and hand, foot and mouth disease in mainland china a systematic review and meta analysis. Iran J Public Health. (2021) 509:1757–65. 10.18502/ijph.v50i9.7046 34722370 PMC8542837

[33] Li T Yang Z Di B Wang M. Hand-foot-and-mouth disease and weather factors in Guangzhou, southern China. Epidemiol Infect. (2014) 142:1741–50. 10.1017/S0950268813002938 24267476 PMC9151230

[34] Yan S Wei L Duan Y Li H Liao Y Lv Q. Short-term effects of meteorological factors and air pollutants on hand, foot and mouth disease among children in Shenzhen, China, 2009-2017. Int J Environ Res Public Health. (2019) 16:3639. 10.3390/ijerph16193639 31569796 PMC6801881

