# Corrigendum: Effects of meteorological factors and atmospheric pollution on hand, foot, and mouth disease in Urumqi Region

**DOI:** 10.3389/fpubh.2023.1358263

**Published:** 2024-01-09

**Authors:** Fang-rong Ren, Yakup Abodurezhake, Zhe Cui, Miao Zhang, Yu-yu Wang, Xue-rong Zhang, Yao-qin Lu

**Affiliations:** ^1^College of Economics and Management, Nanjing Forestry University, Nanjing, China; ^2^College of Public Health, Xinjiang Medical University, Urumqi, China; ^3^Economics and Management School, Nantong University, Nantong, China; ^4^Department of Infectious Disease Control, Urumqi Center for Disease Control and Prevention, Ürümqi, China

**Keywords:** meteorological factor, atmospheric pollution, multiple regression model, prevention of HFMD, communicable disease control

In the published article, there was an error in the correspondence section. Yao-qin Lu should have been listed as the corresponding author. The correct correspondence appears below.

Correspondence: Yao-qin Lu, lyq_superior@163.com

In the published article, there was an error regarding the affiliation(s) for Yakup Abodurezhake. As well as having affiliation(s) 2, they should also have *College of Public Health, Xinjiang Medical University, Urumqi, China*.

In the published article, there was an error in the author list, and author Yakup Abodurezhake was erroneously excluded. The corrected author list appears below.

Fang-rong Ren^1†^, Yakup Abodurezhake^2†^, Zhe Cui^3†^, Miao Zhang^3^, Yu-yu Wang^3^, Xue-rong Zhang^3^ and Yao-qin Lu^4*^

In the published article, there was an error in the Citation. Ren F-r, Cui Z, Zhang M, Wang Y-y, Zhang X-r and Lu Y-q (2022).

The correct Citation statement appears below.

**Citation:** Ren F-r, Abodurezhake Y, Cui Z, Zhang M, Wang Y-y, Zhang X-r and Lu Y-q (2022)

In the published article, there was an error in the Copyright statement. The statement was incorrectly written as “Copyright © 2022 Ren, Cui, Zhang, Wang, Zhang and Lu”. The corrected statement is “Copyright © 2022 Ren, Abodurezhake, Cui, Zhang, Wang, Zhang and Lu. This is an open-access article distributed under the terms of the Creative Commons Attribution License (CC BY). The use, distribution or reproduction in other forums is permitted, provided the original author(s) and the copyright owner(s) are credited and that the original publication in this journal is cited, in accordance with accepted academic practice. No use, distribution or reproduction is permitted which does not comply with these terms.”

In the published article, there was an error in the Author contributions. ZC: conceptualization, investigation, visualization, and supervision. Y-qL: methodology and data curation. F-rR: software, resources, project administration, and funding acquisition. X-rZ: validation and writing—review and editing. MZ and Y-yW: formal analysis. MZ: writing—original draft preparation. All authors read and contributed to the manuscript.

The correct **Author contributions** appears below.

## Author contributions

ZC: visualization, and supervision. Y-qL: methodology and data curation. F-rR: software, resources, project administration, and funding acquisition. YA: conceptualization, investigation and editing. X-rZ: validation and writing—review. MZ and Y-yW: formal analysis. MZ: writing—original draft preparation. All authors read and contributed to the manuscript.

The authors apologize for these errors and state that this does not change the scientific conclusions of the article in any way. The original article has been updated.

